# Genetic architecture modulates diet-induced hepatic mRNA and miRNA expression profiles in Diversity Outbred mice

**DOI:** 10.1093/genetics/iyab068

**Published:** 2021-07-14

**Authors:** Excel Que, Kristen L James, Alisha R Coffey, Tangi L Smallwood, Jody Albright, M Nazmul Huda, Daniel Pomp, Praveen Sethupathy, Brian J Bennett

**Affiliations:** 1 Western Human Nutrition Research Center, Agricultural Research Service, US Department of Agriculture, Davis, CA 95616, USA; 2 Department of Nutrition, University of California, Davis, Davis, CA 95616, USA; 3 Curriculum in Genetics and Molecular Biology, University of North Carolina at Chapel Hill, Chapel Hill, NC 28081, USA; 4 Nutrition Research Institute, University of North Carolina at Chapel Hill, Kannapolis, NC 28081, USA; 5 Department of Genetics, University of North Carolina at Chapel Hill, Chapel Hill, NC 27599, USA; 6 Department of Biomedical Sciences, College of Veterinary Medicine, Cornell University, Ithaca, NY 14853, USA

**Keywords:** quantitative trait loci, multiparental models, eQTL, mirQTL, High-fat diet, High-protein diet

## Abstract

Genetic approaches in model organisms have consistently demonstrated that molecular traits such as gene expression are under genetic regulation, similar to clinical traits. The resulting expression quantitative trait loci (eQTL) have revolutionized our understanding of genetic regulation and identified numerous candidate genes for clinically relevant traits. More recently, these analyses have been extended to other molecular traits such as protein abundance, metabolite levels, and miRNA expression. Here, we performed global hepatic eQTL and microRNA expression quantitative trait loci (mirQTL) analysis in a population of Diversity Outbred mice fed two different diets. We identified several key features of eQTL and mirQTL, namely differences in the mode of genetic regulation (*cis* or *trans*) between mRNA and miRNA. Approximately 50% of mirQTL are regulated by a *trans*-acting factor, compared to ∼25% of eQTL. We note differences in the heritability of mRNA and miRNA expression and variance explained by each eQTL or mirQTL. In general, *cis*-acting variants affecting mRNA or miRNA expression explain more phenotypic variance than *trans*-acting variants. Finally, we investigated the effect of diet on the genetic architecture of eQTL and mirQTL, highlighting the critical effects of environment on both eQTL and mirQTL. Overall, these data underscore the complex genetic regulation of two well-characterized RNA classes (mRNA and miRNA) that have critical roles in the regulation of clinical traits and disease susceptibility

## Introduction

The advent of genome-wide investigation of DNA variants and gene expression has revolutionized our understanding of biology. Systems genetic approaches often utilize variation in DNA and RNA to identify genes and pathways associated with clinical traits (Rockman and Kruglyak 2006). These approaches have been used in studies of plants (Fu *et al.* 2009), flies (Ayroles *et al.* 2009), yeast (Storey *et al.* 2005), mice (Doss *et al.* 2005), and humans (Schadt *et al.* 2003, 2008). Analyzing variation in gene expression in a segregating population for genetic regulation is a critical aspect of systems genetics. Originally, genomic positions that were found to regulate quantitative traits were deemed quantitative trait loci (QTL); correspondingly, loci that regulate the messenger RNA (mRNA) transcript levels are called expression quantitative trait loci (eQTL). QTL and eQTL analyses have identified numerous candidate genes for obesity, diabetes, and cardiovascular disease, indicating that certain genetic variants interact critically with environmental factors that predispose an organism to disease.

The initial studies investigating eQTL were performed in model organisms and the identified eQTL were split into two distinct classes based on the location of the associated single nucleotide polymorphism (SNP). These classes were defined as *cis*-eQTL and *trans*-eQTL based on whether the SNP resided close to or far from the regulated mRNA’s gene of origin. In the case of a *cis*-eQTL, a genomic variant located in proximity to the gene in question is associated with the gene’s expression. The associated SNP is often thought to be in linkage disequilibrium (LD) with the functional genomic variant affecting gene expression. For *cis*-eQTL a number of possibilities exist, such as promoter and enhancer variants. *Trans*-eQTL refer to SNPs associated with gene expression that are located distal to the locus containing the gene in question. *Trans*-eQTL can be located on a different chromosome or at a distal location on the same chromosome, and suggest an alternative regulatory mechanism. More recently, many groups have characterized eQTL and the genetic architecture of gene expression in humans. Several important findings have emerged, including the discovery that *cis*-eQTL, sometimes referred to as “local eQTL,” are pervasive and often enriched for known genome-wide association study (GWAS) loci (Civelek *et al.* 2017). *Trans* eQTL, also referred to as “distant eQTL,” have been found to affect hundreds of genes, are often regulated by the same transcription factors (Brynedal *et al.* 2017), and at times may be transcription factors themselves (Albert *et al.* 2018). In addition, there may be complex relationships between *cis*-acting and *trans*-acting eQTL, as there is evidence that SNPs regulating genes as *cis*-eQTL can also mediate the effects of *trans*-eQTL (Yao *et al.* 2017).

eQTL studies have also begun to investigate the genetic regulation of noncoding RNA. MicroRNAs (miRNAs) are noncoding RNAs that regulate gene expression at the posttranscriptional level and have been implicated in a range of diseases, including cardiovascular diseases and metabolic syndromes (Rottiers and Naar 2012). The identification of microRNA expression QTL (mirQTL) has complemented GWASs and further emphasizes the complex genetic regulation of both miRNA and mRNA. For example, recent data suggest that miRNA are associated with cardiometabolic traits, and Mendelian randomization analysis has demonstrated that these miRNA associations may be causal (Nikpay *et al.* 2019). Determining the underlying mechanisms of eQTL and mirQTL, the interaction of these two classes of RNA, and how eQTL and mirQTL respond to environmental perturbation remains to be fully elucidated.

Human GWAS have been impressive in their ability to associate variants with disease traits, but are limited in their ability to quantify and control environmental effects, their ability to probe tissues, and the sample sizes needed for robust power. To address these limitations, model organisms can be used to identify eQTL and mirQTL in multiple tissues, or under a variety of conditions, such as diet, associating the results with clinical traits and disease susceptibility (Mehrabian *et al.* 2005; Bhasin *et al.* 2008; Yang *et al.* 2009).

Recently, genetic mapping panels have been developed which incorporate variation from more than two parental strains. Using complex breeding strategies, these multiparent advanced generation intercross panels have high genetic resolution and have been employed in a number of model organisms, including *Arabidopsis* (Kover *et al.* 2009), *Drosophila* (Mackay *et al.* 2012), and mice (Aylor *et al.* 2011). In mice, two related multiparent advanced generation intercross populations exist, the Collaborative Cross (CC) and the Diversity Outbred (DO), and both are generated from eight mouse strains, of which five are classical inbred (A/J, C57BL/6J, 129S1/SvImJ, NOD/LtJ, and NZO/HlLtJ) and three are wild-derived inbred (CAST/EiJ, PWK/PhJ, and WSB/EiJ) strains. In comparison to traditional approaches in mice, the inclusion of the wild-derived strains increases genetic diversity and reduces identical-by-descent “blind spots” (Yang *et al.* 2007). The primary difference between CC and DO populations is that the CC is a recombinant inbred panel while the DO is maintained as an outbred population using a randomized breeding scheme. The DO contains ∼45 million SNPs (Yang *et al.* 2011; Churchill *et al.* 2012) and displays a wide variation in phenotypes mimicking the variation observed in humans. Because of the DO population’s allelic diversity, the DO have been utilized in high-resolution genetic mapping of complex traits, including atherosclerosis (Smallwood *et al.* 2014), response to toxicants (French *et al.* 2015), the microbiome (Kemis *et al.* 2019), and diabetes (Keller *et al.* 2018), as well as studies focused on gene expression (Munger *et al.* 2014; Tyler *et al.* 2017), metabolomics, and proteomics (Chick *et al.* 2016). Collectively, these studies utilizing DO mice have provided additional insight into the complex genetic architecture that underlies clinical traits and disease susceptibility (Svenson *et al.* 2012; Chesler *et al.* 2016; Winter *et al.* 2017).

Little is known about the genetic regulation of hepatic mRNA and miRNA under different dietary conditions; exploring these trends in the liver is highly relevant due to the liver's roles in cardiometabolic diseases. We have previously identified novel interactions between eQTL and clinical traits in the DO, including atherosclerosis (Smallwood *et al.* 2014), the atherosclerosis-associated plasma metabolite trimethylamine N-oxide (Coffey *et al.* 2019), and plasma cystatin C (Huda *et al.* 2020). In this study, we investigate the global regulation of hepatic mRNA and miRNA expression in DO mice fed two different diets: a high-fat cholic acid (HFCA) diet designed to trigger atherosclerosis, and a high-protein (HP) diet. We identify eQTL and mirQTL, classify them as *cis* or *trans*, and characterize the QTL by defining heritability, genetic resolution, and effect size in multiple QTL models. Finally, we assess the effect of diet on each of these components and identify eQTL driven by different founder alleles as a result of diet.

## Materials and methods

### Experimental animals and diets

**Figure 1:**
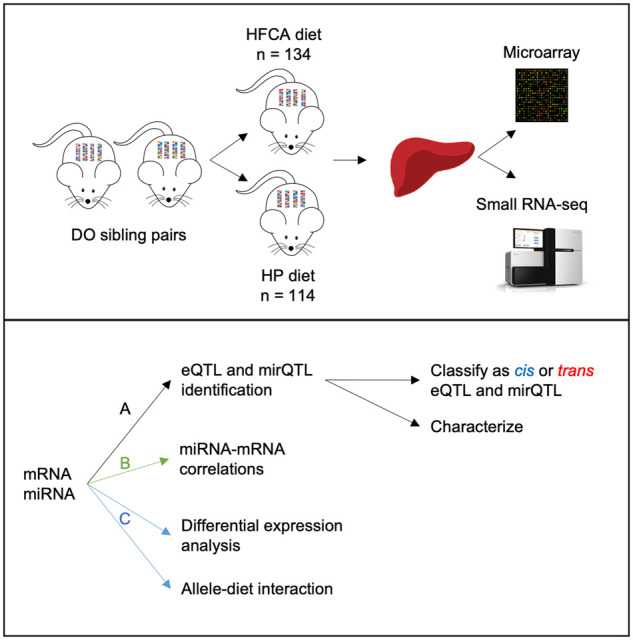
Experimental and analytical design classifies, characterizes, and explores the relationships between hepatic mRNA and miRNA in the context of diet. A total of 292 female DO mice were obtained from Jackson Laboratory as 146 full sibling pairs. After initial maintenance on a synthetic diet (AIN-76A), one sibling from each pair was randomly assigned either an HFCA or HP diet for 18 weeks. Extracted hepatic mRNA was hybridized to the Affymetrix Mouse Gene array and miRNA was sequenced via Illumina HiSeq. mRNA and miRNA expression values were used as phenotypes in four QTL genome scans. (A) The four models were diet as an additive covariate (*n *=* *243), diet as an interactive covariate (*n *=* *243), subset of HFCA-fed mice (*n *=* *134), and subset of HP-fed mice (*n *=* *109). eQTL and mirQTL found in each model were classified as *cis* or *trans* and characterized. (B) Interactions between miRNA and mRNA were assessed and compared between models. (C) The role of diet was explicitly analyzed by differential expression analysis and by identifying eQTL with significant allele-diet interactions from regression analysis.

Details of the mouse experiments have been reported previously (Smallwood *et al.* 2014; Coffey *et al.* 2017, 2019). In brief, 292 female DO mice (J: DO, Jackson Laboratory stock number 009376, outbreeding generation 11) were obtained from the Jackson Laboratory (Bar Harbor, ME) as 146 full sibling pairs at 4 weeks of age. Mice were housed in groups of five per cage in a HEPA-filtered, climate-controlled, facility under a 12-h light–dark cycle and provided with nonirradiated pine bedding and free access to sterile water. Mice were maintained on defined synthetic diet, AIN-76A, until 6 weeks of age, to control for variability in the components of standard chow (D10001; Research Diets, New Brunswick, NJ). Afterward, one sibling from each of the 146 sibling pairs was randomly assigned to one of the diets for a total of 18 weeks (Figure 1). Thus, 146 mice were transferred to a synthetic HFCA diet composed of 20.0% fat, 1.25% cholesterol, and 0.5% cholic acid, to induce atherosclerotic lesions, while the remaining 146 mice were transferred to a nonatherogenic HP diet composed of 5.0% fat and 20.3% protein (D12109C and D12083101, respectively; Research Diets). All procedures were approved by the Institutional Animal Care and Use Committee at the University of North Carolina at Chapel Hill (protocol number 11-299).

### RNA isolation

Livers were flash-frozen in liquid nitrogen and subsequently stored at −80°C until total RNA was isolated using Norgen Total RNA Purification Kit (Norgen, ON, Canada). RNA integrity was determined by Bioanalyzer (Bio-Rad, Hercules, CA, USA) and high- quality RNA (with RNA Quality Index >7.5) isolated from livers of 268 of the 292 DO mice was processed and hybridized to Affymetrix Mouse Gene 2.1 ST 96-Array Plate (Thermo Fisher Scientific, Waltham, MA, USA) using the GeneTitan Affymetrix instrument, according to the standard manufacturer’s protocol. All probes containing known SNPs from the eight founder inbred mouse strains of the DO mouse population were masked (165,204 probes) during normalization by downloading the SNPs from the Sanger sequencing website (http://www.sanger.ac.uk/science/data/mouse-genomes-project) and overlapping them with probe sequences. To ensure integrity of downstream qualitative analyses, annotation data from the Affymetrix mogene 21 annotation database, Bioconductor version, release 3.7 (http://www.bioconductor.org/packages/release/data/annotation/html/mogene21sttranscriptcluster.db.html) was used to filter the remaining transcript cluster IDs for those with reliable and complete annotations. We removed transcript cluster IDs identified as cross-hybridizing (*n *=* *4954), associated with unlocalized sequences (*e.g.*, chr1_GL456210_random), residing on the *Y* chromosome or mitochondria (*n *=* *69), or without ENSEMBL or ENTREZ annotations (*n *=* *4766). The total number of unique probes postfilter was 24,004, corresponding to 23,626 genes. All transcript cluster IDs were validated by programmatically querying the ENTREZ IDs against the NCBI Gene Database for chromosome and position. Microarray data are available on the Gene Expression Omnibus repository under accession number GSE99561.

### Small RNA sequencing

High-quality RNA from livers of the DO mice was also used for small RNA sequencing (smRNA-seq). Libraries were created using NEBNext Multiplex Small RNA Library Prep Set for Illumina (New England Biosciences), and 50-bp single-read sequencing was carried out on the Illumina HiSeq platform, resulting in an average of over 16 million reads per sample. miRquant 2.0 (Kanke *et al.* 2016) was used to trim off adapter sequences, align reads to the mouse genome, and quantify miRNAs and their isoforms (termed isomiRs). A previous study in mice from the CC mouse panel has shown that miRNAs do not contain variants across founder strains within their seed regions, so reads were aligned to the mm9 mouse genome (Rutledge *et al.* 2015). Reads were normalized to reads per millions mapped to miRNAs (RPMMMs). An expression threshold of at least 50 RPMMMs in about one-quarter of all samples was set to filter out the lowly expressed miRNAs, which resulted in a set of 246 robustly expressed miRNAs.

### Genotyping

DNA was extracted and purified from tail biopsies taken from 6-week-old mice using the QIAGEN DNeasy kit, following the manufacturer’s instructions. Genotyping was performed using the Mega Mouse Universal Genotyping Array (MegaMUGA) by GeneSeek (Neogen, Lincoln, NE, USA) (Svenson *et al.* 2012).

### QTL mapping

QTL mapping was performed using the R (v3.5.1) package QTL2 (v0.20). The genotypes from the MegaMUGA array are assigned DO founder strain probabilities using a hidden Markov model (Broman and Sen 2009) and haplotypes are defined as previously reported (Broman 2012a, 2012b). The genotype probabilities are reduced to the eight founder allele probabilities and used to generate a kinship matrix using the “leave-one-chromosome-out” method to reduce bias from same chromosome SNPs (Yang *et al.* 2014). A genome scan is performed using a linear mixed model, which regresses the microarray gene expression phenotype matrix against the allele probabilities at each marker, using the kinship matrix to control for population structure.

Using the scan1 function in R/qtl2, four separate genome scans were performed to assess how genotype, diet, and gene-by-diet interactions affect eQTL. The four QTL models are as follows: diet as an additive covariate, diet as an interactive covariate, subset of mice fed an HFCA diet (*n *=* *134), and subset of mice fed an HP diet (*n *=* *109). The phenotypes for the genome scans were the individual expression data for each transcript cluster ID. To assess similar effects on mirQTL, the same models were performed on the miRNA abundance data. The log of the odds ratio (LOD), which describes the log-scaled likelihood difference of the full and null genome scan models, is generated for each SNP on the MegaMUGA (*n *=* *70,339) genotyping array. Permutation analysis was used for subsequent filtering (described below), reducing the initial output to a set of high-confidence QTL. Confidence intervals for QTL were calculated by determining the upper and lower physical distances from the peak where the LOD dropped by 1.8 or more, approximating a 95% coverage of true positives for an intercross population (Manichaikul *et al.* 2006). We defined eQTL as *cis*-eQTL if the marker with the maximum LOD score was within 4 Mb of the transcription start site (TSS), and called the remaining eQTL *trans*-eQTL (Keller *et al.* 2018).

### Permutation analysis

The significance threshold at *P *<* *0.05 of each QTL was empirically determined via permutation analysis to minimize type 1 and type 2 errors (Huda *et al.* 2020). The rows of the genotype data were randomized with respect to the expression data (miRNA or mRNA) and a maximum LOD score was produced. This process was repeated 1000 times to create a null distribution of maximum LOD scores for a particular expression trait (Churchill and Doerge 1994). The 95th quantile of a phenotype’s null distribution was considered as its individual significance threshold at *P *=* *0.05. For our analysis with diet as an additive covariate, all transcripts (*n *=* *24,004) were subjected to full permutation analysis.

A modified approach, designed to reduce the computational complexity of performing 1000 QTL permutations analysis on 24,004 transcripts three times, was utilized to identify eQTL for the interactive and diet-specific subsets. After the initial eQTL model was fit for each transcript, we completed 50 permutations to generate an initial null distribution for each candidate, calculating a conservative threshold by taking the 90th quantile and subtracting the quantile SE (Cox and Hinkley 1979). The transcripts with LOD scores above this threshold (90th quantile minus the quantile SE) were subjected to full 1000 permutation testing. Transcripts with LOD scores above the 95% significance threshold, as determined by 1000 permutations, were considered significant.

We initially validated this method with diet as an additive covariate, which had 1000 permutations performed for each transcript cluster ID. Using the 50-permutation method, we found 8867 transcripts to be above our modified threshold. We subjected these 8867 transcripts to permutation testing (1000 permutations) and identified the same transcripts with significant eQTL (*n *=* *6276) as our initial test of all 24,004 transcripts.

### Genome scan coefficients

The contribution of each founder strain genotype at each QTL was determined using the best linear unbiased predictor (BLUP) for each QTL model (additive, HFCA diet, and HP diet) using a similar mixed-effect model that treated the allele probabilities as random effects. BLUP scans show contributions from the eight DO founder strains at each SNP and are extracted from the peak SNP of each significant eQTL or mirQTL.

### Phenotypic variance explained

For each significant eQTL or mirQTL, the RNA expression and genotype probabilities at the peak SNP were extracted and fit to full and null Haley–Knott regression models. The differences in *R*^2^ values between the full model, with genotypes and covariates included, and null model with only covariates included, were taken as the phenotypic variance explained by the QTL.

### Heritability

To determine the extent to which phenotypic variation is influenced by genotypic variation, we used a linear mixed effect model to estimate narrow-sense heritability (*h*^2^) scores of transcript cluster IDs or miRNAs. This was performed using the function est_herit in R/qtl2, submitting a single, square, kinship matrix, and the expression value of individual transcript cluster IDs or miRNA with eQTL or mirQTL.

### Enrichr

Using the Ma’ayan Laboratory’s Enrichr Tool (https://amp.pharm.mssm.edu/Enrichr/), we used the httr R library to programmatically submit gene sets to all gene-set libraries available on Enrichr via its Application Programming Interface (https://amp.pharm.mssm.edu/Enrichr/help#api). We then filtered the results to only include those with adjusted *P *<* *0.05.

### Correlation patterns in miRNA and mRNA

Pairwise Spearman correlation was performed on every sequenced miRNA (*n *=* *246) and annotated mRNA probe (*n *=* *24,004) pair. The outputs were combined and filtered for significance at a Benjamini–Hochberg (BH)-adjusted *P *<* *0.05, resulting in 1,408,235 mRNA-miRNA correlations. Correlations were divided by mRNA into three categories based on the given model eQTL results: mRNA with significant eQTL were subdivided into “*cis*” and “*trans*,” and mRNA with no significant eQTL were designated “no eQTL.” To better assess the differences between *cis*- and *trans*-eQTL, mRNA with multiple mappings (additive = 421; HP diet = 180; HFCA diet = 235) were removed for this analysis. Pairwise Wilcoxon rank-sum tests were performed on the absolute values of Spearman’s rho for the three groups. Fisher’s exact test was performed to test for dependence of mapping status and known miRNA-mRNA interactions. The *Mus musculus* miRNA catalog was downloaded from miRTarBase release 7.0 (Chou *et al.* 2018), and used to identify known miRNA-gene interactions.

### mRNA eQTL and miRNA mirQTL colocalization

The confidence intervals of each eQTL and mirQTL on the same chromosome were tested for overlap. For those eQTL- mirQTL pairs with overlapping confidence intervals, a physical distance between peak markers was calculated. Pairs with a distance of zero were mapped to the same SNP.

### Differential gene expression

To determine the effect of diet on hepatic mRNA and miRNA, we performed differential gene expression analysis. Transcripts with a robust multiarray average value of four or greater in at least 25% of samples were included (Coffey *et al.* 2017), which resulted in 11,370 transcripts and 246 miRNA. The Wilcoxon rank-sum test was performed for both miRNA and mRNA data and *P*-values were corrected using the BH method. Significance was set at *P* < 0.05.

### Allele-diet interaction identification

The interactive model generated using R/qtl2 suggested that many eQTL have unique diet effects from permutation analysis of LOD scores, but did not allow us to identify eQTL with a significant allele-by-diet interaction term within the model. To achieve this, we performed an ANOVA of the allele-diet interaction against the expression of the transcript with a significant eQTL in the interactive model. Interactive eQTL with a significant allele-diet interaction term (BH-adjusted *P* < 0.05) were subset to eQTL present in both the HFCA and HP diet models. For this subset, we calculated the founder allele effects using the R/qtl2 BLUP scan method in each diet model. We calculated the percent difference of founder effects of the HFCA diet model, using HP diet as a reference, at each eQTL. Finally, we summed the absolute value of the change across all of the founders for each gene, identifying those whose founder effects are most variable as a result of diet.

### Data availability

Microarray and smRNA-seq data are available on the Gene Expression Omnibus repository under accession number GSE99561. Genotypes are available at DRYAD: https://doi.org/10.25338/B87K75. Supplemental material includes all significant QTL identified in this study. Supplementary material is available at figshare: https://doi.org/10.25386/genetics.12597794.

## Results

### Genetic architecture of hepatic mRNA and miRNA expression vary within the DO population

**Figure 2:**
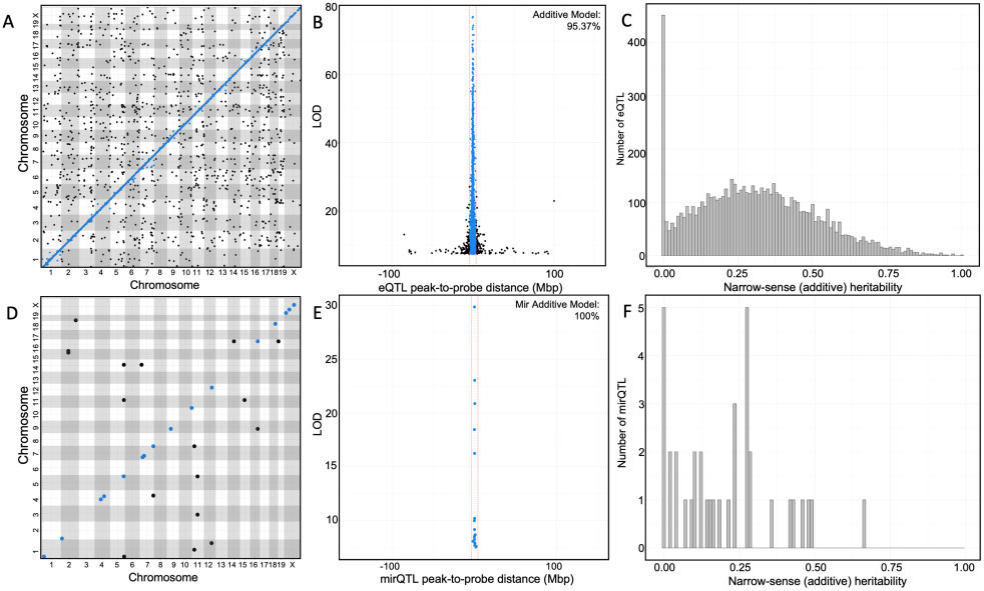
High-resolution hepatic eQTL and mirQTL in Diversity Outbred mice demonstrate complex regulation of expression traits. Results from expression quantitative trait loci (eQTL) analyses using a genome scan model treating the HFCA diet and HP diet groups as an additive covariate. (A) An eQTL plot visualizes all significant associations between gene expression and structural SNP variants from a genome scan. The absolute genomic positions of the SNP and gene transcript probe set in the mouse genome are shown on the *x*- and *y*-axes, respectively. The blue line represents *cis*-eQTL, positions where gene expression variance is associated with a proximal (±4 Mb) SNP variant. (B) Resolution of *cis*-eQTL is determined by the distance from the SNP to the transcript probe set (*x*-axis); resolution across all 6183 eQTL-probe pairs on the same chromosome shows 95.4% of *cis*-eQTL are within ±4 Mb of their probe set and tend to have highly significant log of the odds scores (LOD, *y*-axis). *Cis*-eQTL resolution in the DO population is estimated to be 0.30 Mb. (C) Narrow-sense heritability is calculated for the probe set expression of each significant eQTL in the additive QTL analysis. (D) mirQTL plot of the results from the genome scan of miRNA transcription data with diet as an additive covariate. (E) Resolution of 19 *cis*-mirQTL, showing 89.5% of *cis*-mirQTL are within ±4 Mb. *Cis*-mirQTL resolution in the DO population is estimated to be 0.23 Mb. (F) Distribution of narrow-sense heritability for the miRNA expressions with significant mirQTL in the additive genome scan.

To identify the global regulation of hepatic gene expression, we performed a genome scan on all 243 female HFCA- or HP-fed DO mice (134 HFCA-fed and 109 HP-fed), treating diet as an additive covariate. We identified eQTL and mirQTL in the full DO cohort. For eQTL, the median LOD score that corresponded to the 95% of genome-wide type 1 error rate was 7.46 (Table 1). The distribution of LOD scores significant at *P *<* *0.05 is shown in Supplementary Figure S1. Using permuted LOD thresholds for individual transcripts, we identified a total of 7696 significant eQTL, representing 7202 unique transcript cluster IDs that map to 7118 unique genes (Figure 2A). We next identified the 6183 eQTL associated with SNPs residing on the same chromosome as the gene probe and calculated the distance between the SNP and the gene’s TSS. The mean absolute distance between the TSS and the peak SNP’s physical location was 1.21 Mb and the median distance was 0.30 Mb (Figure 2B). To allow for variable recombination rates across the genome, we used a genomic interval of 4 Mb to classify transcripts as high-confidence *cis*-eQTL. Using this metric, we classified 5603 eQTL as *cis*-eQTL (Supplementary Table S1). To support the decision to classify transcripts using a 4-Mb genomic interval, we tested a 1-Mb interval, which produced similar distributions of *cis* and *trans* due to the high resolution afforded in the DO (Supplementary Table S2); therefore, all subsequent analyses were performed using the 4-Mb interval.

**Table 1:** Means and ranges of permutation thresholds by significance level

	Additive model		Interactive model		High-fat cholic acid diet		High-protein diet	
	Mean	Range	Mean	Range	Mean	Range	Mean	Range
mRNA								
*P *<* *0.05	7.47	6.88–9.21	10.83	10.09–18.61	7.65	6.98–10.02	7.72	7.01–12.99
*P *<* *0.63	6.18	5.77–6.74	9.27	8.68–14.22	6.35	5.76–7.11	6.39	5.89–8.32
miRNA								
*P *<* *0.05	7.66	7.31–8.44	11.36	10.56–14.11	7.96	7.56–9.16	8.37	7.56–15.02
*P *<* *0.63	6.24	6.13–6.56	9.54	9.05–10.70	6.46	6.30–6.88	6.62	6.36–8.60

We treated all 2093 eQTL that did not fall into the *cis*-eQTL category as *trans*-eQTL, where a distal SNP (either >4 Mb on the same chromosome or on a different chromosome) is regulating the expression of a gene (Figure 2A; Table 2). When the position of the peak QTL SNP was plotted against the QTL probe position, we observed several vertical lines that may represent *trans*-eQTL bands. A total of 483 transcript cluster IDs exhibited multiple mappings, 62 of these *trans*-eQTL were the only genetic regulation observed, while 421 had both *cis*- and *trans*-eQTL signals (Table 3). All significant *trans*-eQTL are shown in Supplementary Table S3.

**Table 2:** Summary of eQTL results by model

Run^a^	Additive model	Interactive model	High-fat cholic acid diet	High-protein diet
mRNA				
*Cis* (% total)	5,603 (72.8)	4,861 (69.1)	3,584 (64.0)	3,254 (65.1)
*Trans* (% total)	2,093 (27.2)	2,171 (30.9)	2,012 (36.0)	1,748 (34.9)
Total	7,696	7,032	5,596	5,002
miRNA				
*Cis* (% total)	17 (56.67)	12 (54.55)	9 (47.37)	9 (30.00)
*Trans* (% total)	13 (43.33)	10 (45.45)	10 (52.63)	21 (70.00)
Total	30	22	19	30

^a^ *Cis* and *trans* designations are defined as QTL peaks whose probes are within ± 4 Mb of the starting site or not, respectively.

**Table 3:** Summary of eQTLs with multiple mappings by model

Run^a^	Additive model	Interactive model	High-fat cholic acid diet	High-protein diet
mRNA				
Mixed (% total)	421 (87.16)	356 (79.11)	235 (76.55)	180 (76.27)
*Trans* only (% total)	62 (12.84)	94 (20.89)	72 (23.45)	56 (23.73)
Total	483	450	307	236
miRNA				
Mixed (% total)	0 (0)	1 (100)	0 (0)	0 (0)
*Trans* only (% total)	1 (100)	0 (0)	0 (0)	1 (100)
Total	1	1	0	1

^a^ mRNA probes that appear as eQTLs at multiple locations on the genome have multiple mappings. There were no multiple mapping eQTLs that all mapped to the same chromosome (*cis* only), all others were a mix of *cis* and *trans* (mixed) or only *trans* (*trans* only).

We then identified mirQTL in the full DO cohort, treating diet as an additive covariate, and determined the threshold of significance by performing 1000 permuted genome scans on all 246 robustly expressed miRNAs. The median LOD score for a *P *<* *0.05 threshold was 7.54. We identified a total of 30 miRNA with significant mirQTL (Figure 2D). From these, we identified 19 miRNAs whose peak eQTL was regulated by a SNP residing on the same chromosome as the miRNA’s physical position. This distance ranged between 16.64 and 21.473 Mb, with median resolution of 0.23 Mb, which corresponds approximately to the same resolution calculated for mRNA (0.30 Mb) and allows us to classify 17 as *cis*-mirQTL using the 4 MB definition. The *cis*-mirQTL are shown in Supplementary Table S4 and *trans*-mirQTL are shown in Supplementary Table S5.

Notably, the distribution of *trans* and *cis* were different for eQTL and mirQTL, as *cis*-eQTL made up 73% of the eQTL compared to only 57% of the mirQTL. A majority (85%) of *trans*-mirQTL (*n *=* *19) were found on chromosomes different from the physical location of their miRNA (Table 2). We identified one miRNA that had multiple mirQTL.

### Heritability and effect size of eQTL and mirQTL in the DO population

**Figure 3:**
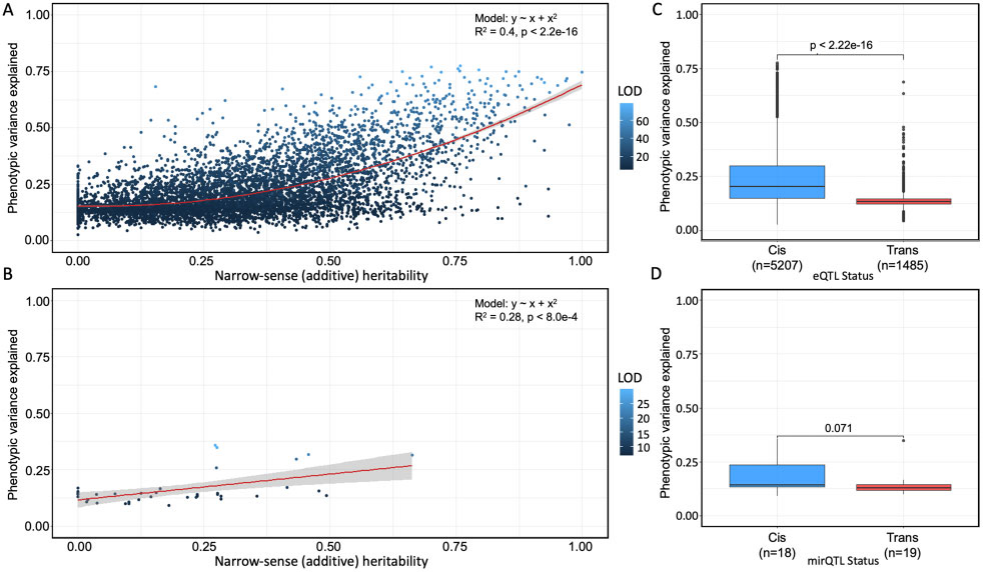
eQTL and mirQTL differ in heritability and effect size in Diversity Outbred mice. (A, B) Narrow-sense heritability scores and *R*^2^ differences between full and null Haley–Knott regression models (phenotypic variance explained) were calculated for every significant eQTL and mirQTL in the additive genome scan models and colored by their LOD score. Polynomial and simple regression models were fit to the mRNA and miRNA data, respectively, to understand the association between heritability (*x*-axis) and phenotypic variance explained by the eQTL (*y*-axis). (C, D) Wilcoxon rank-sum tests reveal a significant difference in the phenotypic variance explained between *cis*- and *trans*-eQTL and a similar but insignificant (*P *≥* *0.05) difference in the phenotypic variance explained between the *cis* and *trans*-mirQTL.

To further explore the genetic architecture of hepatic mRNA and miRNA, we sought to determine the variance explained by each significant eQTL and the narrow-sense heritability (*h*^2^) of each eQTL’s associated gene. Among the genes with eQTL, the heritability ranged between 0.0 and 1.0, with a median *h*^2^ of 0.336 (Figure 2C). The variance of expression levels explained by the peak eQTL SNP ranged between 0.023 and 0.933, with a median of 0.182. There was a significant positive correlation (rho = 0.62, *P *<* *2.2 × 10^−16^) between narrow-sense heritability of gene expression and variance explained by eQTL (Figure 3A). The allele probabilities of the peak SNP associated with *cis*-eQTL explained a median of 21.8% of the variation in gene expression, while the peak SNP associated with *trans*-eQTL explained significantly less variation (Wilcoxon rank-sum, *P *<* *2.2 × 10^−16^), at 13.77% (Figure 3C).

We next assessed the heritability of mirQTL, which was generally less heritable than eQTL. Among the miRNA with mirQTL, the heritability ranged between 0.0 and 0.83, with a median *h*^2^ of 0.24 (Figure 2F). The variance explained by the peak mirQTL SNP ranged between 0.098 and 0.430, with a median of 0.16. Similar to the finding in eQTL, there was also a correlation between narrow-sense heritability of gene expression and variance explained by mirQTL (Figure 3B). The allele probabilities of the peak mirQTL SNPs associated with *cis*-mirQTL explained 16.72% of the variation in gene expression and was significantly less than the 13.77% explained by *trans*-mirQTL (*P *=* *0.0081; Figure 3D).

### Wild-derived alleles influence eQTL and mirQTL

**Figure 4:**
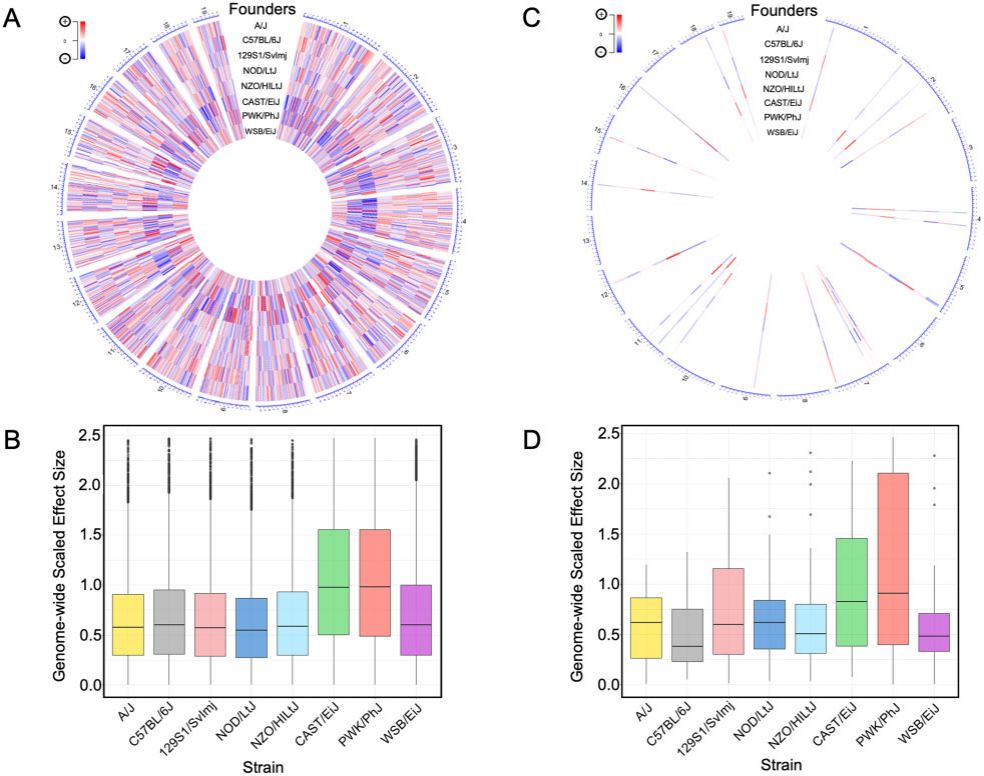
Alleles from wild-derived strains contribute to eQTL and mirQTL. (A) Center-scaled best linear unbiased predictor (BLUP) coefficients from eight DO founder strains are taken at the peak SNP of all significant eQTL from the additive model. Their relative effect sizes are visualized and mapped to the eQTL’s physical location on a circularized mouse genome. The sixth and seventh tracks reveal a clear pattern of peak contribution (dark red and blue bands) from wild-type founder strains CAST/EiJ and PWK/PhJ that is consistent across the genome. (B) Boxplot representation of center-scaled BLUP coefficients from all significant eQTL in the additive model. Kruskal–Wallis rank-sum test indicates significant differences (*P *<* *2.2 × 10^−16^) among founder effects. Dunn’s *post hoc* test confirms significantly (BH-adjusted *P *<* *1.0 × 10^−4^) larger effects from the CAST/EiJ and PWK/PhJ strains relative to all other strains. (C) BLUP coefficients from eight DO founder strains are taken at the peak SNP of all significant mirQTL from the additive model. Their relative effect sizes are visualized and mapped to the mirQTL’s physical location on a circularized mouse genome. Patterns of peak contribution by any particular strain are visually unclear. (D) Box-plot of center-scaled BLUP coefficients from all significant mirQTL in the additive model. Kruskal–Wallis rank-sum test does not indicate significant differences (*P *<* *0.1995) among founder effects.

A significant feature of the DO population is that allele distributions are composed of, on average, 12.5% from each of the eight founder strains at any given locus, meaning 37.5% of the genome is inherited from the three wild-derived founder strains (CAST/EiJ, PWK/PhJ, and WSB/EiJ) (Chesler *et al.* 2016). These three strains contribute a significant portion of the genetic variants in the DO population. Thus, we sought to determine if any of these strains’ individual allele effects disproportionally contribute to eQTL and mirQTL. To do so, we mean-center-scaled the BLUP allele effects for the eight founder strains at the peak SNP of each eQTL (Figure 4A) and mirQTL (Figure 4C). The scaled founder effect sizes from all significant eQTLs demonstrated an enriched signal for alleles contributed by CAST/EiJ and PWK/PhJ strains (Kruskal–Wallis on the effect sizes, *P *<* *2.2 × 10^−16^) (Figure 4B). Dunn’s *post hoc* analysis, with BH multiple comparison corrections, confirmed that CAST/EiJ and PWK/PhJ allele effects were significantly larger than all other founder strains (*P *<* *1.0 × 10^−16^). Testing for this same effect in mirQTL was inconclusive; Kruskal–Wallis one-way ANOVA did not indicate statistically significant differences among founder strain mirQTL contributions (*P *<* *0.069; Figure 4D).

### Characterization of hepatic mRNA and miRNA interactions

We investigated if similar genetic architecture affects hepatic mRNA and miRNA expression by examining eQTL and mirQTL with overlapping confidence intervals (see Materials and Methods). We identified 29 mirQTL whose confidence interval overlapped with the confidence interval of 551 eQTL. The miRNAs were approximately evenly distributed between *cis* and *trans* regulation, with 12 miRNAs being regulated in *trans* and 17 in *cis*. mRNAs were regulated more frequently by local variants as there were 374 *cis*-eQTL and only 177—*trans*-eQTL. The distance between the SNPs regulating mRNA and miRNA averaged 4.86 Mb and ranged between 0 and 112 Mb (Supplementary Table S6). Notably, several mRNA and miRNA were regulated by the same SNP and included cis and trans-eQTL and mirQTL. The functional relevance of having *cis*-eQTL and *cis*-mirQTL co-regulated at the same locus remains to be fully elucidated.

A single miRNA can affect the expression of multiple mRNAs and thus play critical roles in the regulation of gene expression. So, in addition to shared genetic regulation, one can hypothesize that the correlation structure between miRNA and mRNA will result in significant interactions. We correlated expression levels of each miRNA with each mRNA and generated 5,904,492 correlations, of which 1,408,235 were significant after BH multiple comparison correction. The direction of the correlation may inform the biological association between the miRNA and mRNA and we observed a similar number of positive correlations (*n *=* *723,957) and negative correlations (*n *=* *684,278). We next categorized each of the correlations into three categories: miRNA: *cis*- eQTL, miRNA: *trans*-eQTL, and miRNA: no-eQTL. There were 397,290 significant miRNA: *cis*-eQTL correlations, 143,641 significant miRNA: *trans*-eQTL correlations, and 867,304 significant miRNA: no-eQTL correlations (Supplementary Figure S2). The median (±SEM) absolute Spearman’s rho for mRNA with *trans*-eQTL was 0.241 ± 0.00030 and was significantly different from both *cis*-eQTL and no-eQTL (*cis*-eQTL: *P *<* *2.2 × 10^−16^, 0.236 ± 0.00016; no-eQTL: *P *<* *2.2 × 10^−16^, 0.233 ± 0.00012). Although statistically significant, it is difficult to conclude whether the absolute expression of miRNA favors one form of genetic regulation.

Our global analysis of miRNA and mRNA was broad; therefore, we sought to characterize the miRNA-mRNA correlations based on known interactions. Of the 1,408,235 significant correlations, 1895 had validated miRNA-mRNA interactions on miRTarBase (Supplementary Table S7) and 60% were positively correlated. We next characterized these high confidence miRNA-mRNA interactions based on the genetic regulation of the mRNA associated with the miRNA. Fisher’s exact test on the dependence of mapping status (mapping: 540,931; no mapping: 867,304) and validated miRTarBase interaction (interaction: 1905; no interaction: 1,406,330) reveals no significant difference between validated miRNA-mRNA interactions and eQTL with or without an eQTL (odds ratio 0.94, 95% CI 0.86–1.04, *P *=* *0.2294) (Table 4). This finding supports that miRNA-mRNA interactions are observed at similar rates in the presence and absence of a strong ge netic signal.

**Table 4:** Fisher’s exact test reveals no significant difference between significantly correlated pairs of miRNA and mRNA and eQTL status (odds ratio 0.94, 95% CI 0.86111.04, *P *=* *0.2294)

		Interaction	
		Yes	No
Mapping	Yes	706	540,225
	No	1,199	866,105

Fisher’s exact test did not show significant association between known interactions and *cis vs trans* mapping, *P* = 0.11. Fisher’s exact test did not show significant associations between known interactions and *cis vs trans* mapping, *P *=* *0.15.

### Diet has profound effects on the genetic regulation of hepatic mRNA and miRNA expression

Having classified and characterized hepatic mRNA and miRNA while controlling for diet, we next sought to investigate the specific effects of diet on gene and miRNA expression (Figure 1C). We performed differential expression analysis by diet using the mRNA and miRNA that passed the robust multiarray average and RPMMM thresholds (see Materials and Methods). A total of 8657 (76%) mRNA and 196 (80%) miRNA were differentially expressed, indicating a predominant effect of diet on the overall transcriptional profile (Supplementary Tables S8 and S9). We classified the differentially expressed genes by their eQTL status in the additive model and found that 42% had an eQTL. In comparison, only 12% of differentially expressed miRNA had a mirQTL (Table 5). Using Fisher’s exact test, we calculated that mRNA is less likely to have an eQTL when they are differentially expressed (odds ratio 0.81, 95% CI 0.74–0.88, *P *<* *0.001). A stronger but nonsignificant trend was observed with miRNA.

**Table 5:** Fisher’s exact test reveals that mRNA are less likely to have an eQTL when they correspond to a differentially expressed gene (odds ratio 0.81, 95% CI 0.74–0.88, *P *<* *0.001)

	Differential expression			
		**mRNA**		
		**Yes**	**No**	**eQTL**
eQTL	Yes	4,045	1,410	48%
	No	4,612	1,303	52%
		**miRNA**		
		**Yes**	**No**	**mirQTL**
mirQTL	Yes	21	8	12%
	No	172	42	88%

A stronger but nonsignificant trend is observed with miRNA.

We then analyzed the relationship between effect size, or phenotypic variance explained, and differential gene expression for the peak SNP of *cis*- and *trans*-eQTL in the additive model. The median phenotypic variance explained by the peak SNP associated with the *cis*-eQTL for a differentially expressed transcript was 0.20 (range, 0.035–0.81). In contrast, for genes without a statistically significant effect of diet on transcript levels, the median phenotypic variance explained was higher at 0.271 (range, 0.128–0.855). Thus, genes affected by diet had less of their variation explained by the peak SNP associated with an eQTL, underscoring the influence of the environment on this subset.

**Figure 5:**
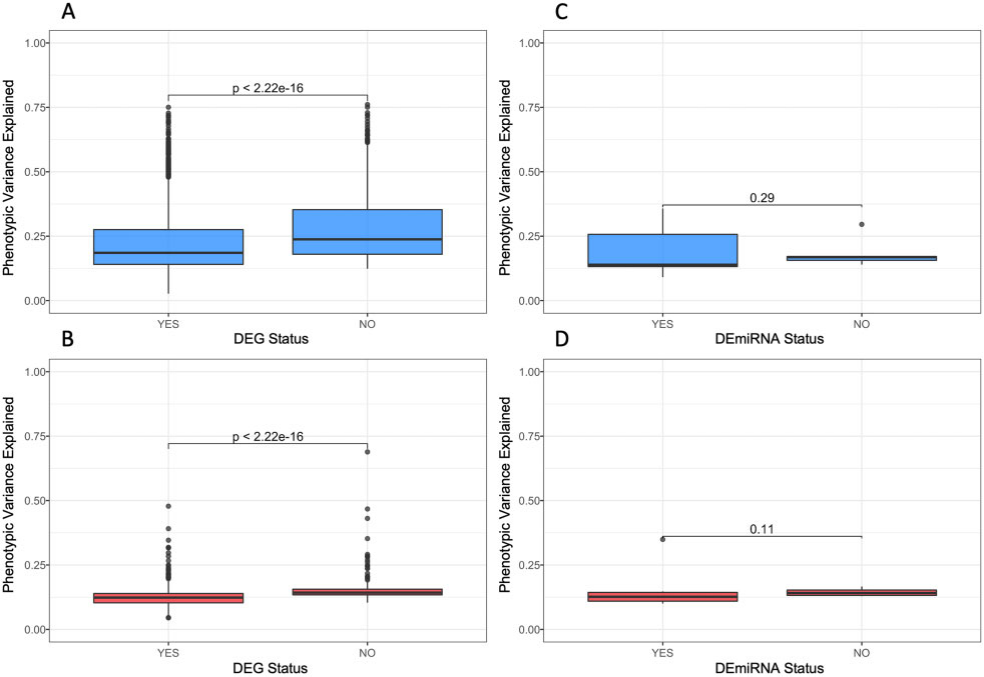
Effect size of eQTL is lower in genes and miRNA that are differentially expressed. The allelic effect size is significantly lower (*P *<* *2.22 × 10^−16^) in genes that are differentially expressed in both *cis*- and *trans*-eQTL. (A) For *cis*-eQTL, the median variance explained when the transcript is differentially expressed is 0.20 compared to 0.246 when it is not differentially expressed. (B) For *trans*-eQTL, the median variance explained when the transcript is differentially expressed is 0.127 compared to 0.142 when it is not differentially expressed. (C) The allelic effect size is not significantly different in *cis* or *trans*-mirQTL; however, the median of differentially expressed *cis*-mirQTL is 0.164 compared to 0.215 when not differentially expressed. (D) Similarly, differentially expressed mirQTL in *trans* had a smaller median than those not differentially expressed at 0.124 and 0.158, respectively.

Furthermore, analysis of the mode of regulation of eQTL (*cis* or *trans*) coupled with the differential expression results paralleled our previous finding that *trans*-eQTL tended to explain less variance than *cis*-eQTL. For differentially expressed transcripts with a *trans*-eQTL, the variance in expression explained by the eQTL was 0.127 (range, 0.039–0.848), whereas the variance for transcripts not affected by diet was greater at 0.143 (range, 0.023–0.804). In summary, for both *cis* and *trans*, the median variance explained was significantly greater for mRNA that were not differentially expressed (Figure 5, A and B). This pattern was also observed for miRNA; however, the results were not significant, which may reflect the relatively low number of mirQTL (Figure 5, C and D).

**Figure 6:**
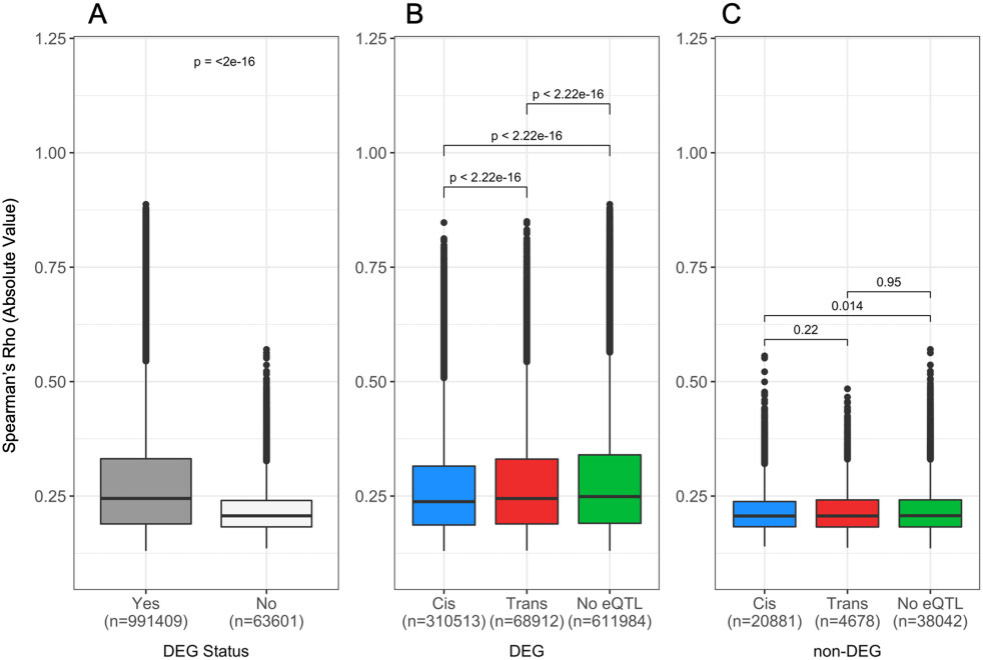
Magnitude of correlation is similar between miRNA and mRNA with *cis*, *trans*, and no-eQTL and reduced when mRNA are not differentially expressed. Expression data were used to generate correlations between pairs of miRNA (*n *=* *246) and mRNA (*n *=* *24,004) and the results were filtered for false-discovery-rate-corrected significance (α ≤ 0.05), resulting in 1,408,235 significant correlations. miRNA-mRNA pairs were classified by their eQTL status in the additive model. A total of 1,055,010 eQTL with transcripts that passed the quality threshold for the DE analysis were included. (A) Spearman’s rho was plotted against the DE status of the mRNA from the additive genome model. Median ± SE for DEG and non-DEG include 0.247 ± 0.0001 and 0.209 ± 0.0002, respectively. (B and C) Spearman’s rho was plotted against the eQTL status of mRNA (B) that were differentially expressed by diet and (C) that were not differentially expressed by diet. For differentially expressed mRNA with *cis*, *trans*, and no-eQTL, the median ± SE are 0.231 ± 0.0002, 0.250 ± 0.0004, and 0.251 ± 0.001, respectively. For nondifferentially expressed mRNA with *cis*, *trans*, and no-eQTL, the median ±SE are 0.208 ± 0.003, 0.210 ± 0.0006, and 0.209 ± 0.003, respectively. DEG, differentially expresses gene.

To further characterize the behavior of differentially expressed genes and miRNA, we returned to our miRNA- mRNA interaction analysis. We assessed the magnitude of the miRNA-mRNA correlation by their mapping (miRNA: *cis*-eQTL, miRNA: *trans*-eQTL, and miRNA: no-eQTL) and differential expression status and found that in all instances, differentially expressed mRNA and miRNA have a statistically higher (*P *<* *2.22 × 10^−16^) absolute rho than their nondifferentially expressed counterparts (Figure 6A). Further classification of the relationship between miRNA and mRNA based on differentially expressed gene status and classification of eQTL indicated that there was a small but statistically significant effect between transcripts with *cis*-eQTL and trans or no-eQTL in both DE conditions (Figure 6, B and C). Overall, the effect of environmental factors such as diet has critically important effects on the relationship between hepatic mRNA and miRNA.

### Diet alone affects *cis*- and *trans*-eQTL and mirQTL architecture

**Figure 7:**
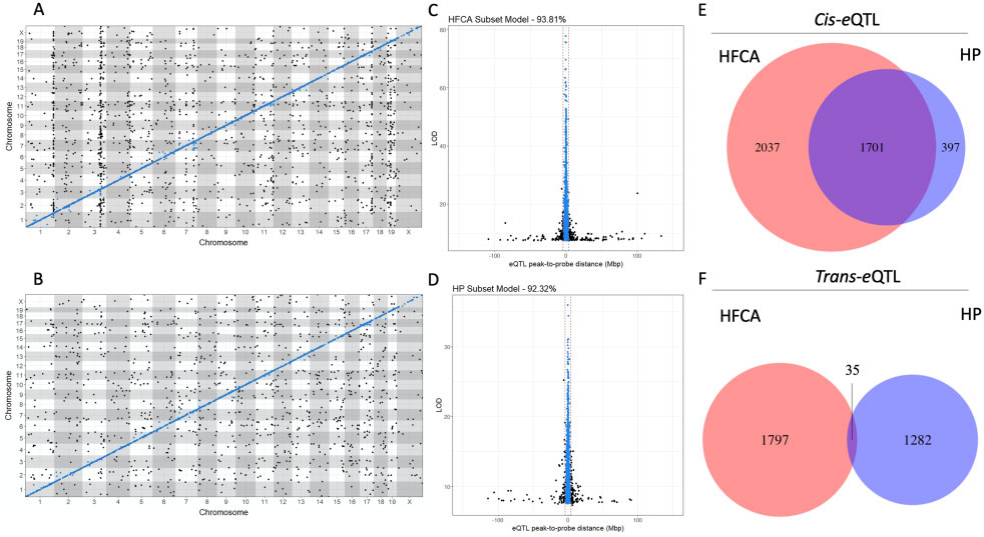
Analysis of genome scans from HFCA- and HP-fed mice reveal environmentally driven differences in genetic architecture. (A) Global eQTL architecture for the HFCA diet model shows numerous, dense *trans*-bands, in contrast to the (B) HP diet model, which shows notably less colocalization of *trans*-eQTL. Both diet models (C and D) show similar precisions from eQTL with probe sets on the same chromosome; resolution of these 4036 eQTL from HFCA-fed mice and 3648 eQTL from HP-fed mice are estimated to be 0.355 and 0.359 Mb, with 88.8 and 89.2% of *cis*-eQTL occurring ±4 Mb from their probe sets, respectively. This indicates that structural variants associated with local genetic regulation tend to occur close to the gene itself, regardless of environmental perturbations. (E, F) A large number of *cis*-eQTL overlap despite environmental differences, while the lack of overlap in the *trans*-eQTL might be indicative of environmental effects.

We have previously reported that diet significantly affects miRNA and mRNA expression in the DO population (Coffey *et al.* 2017). Moreover, having identified relationships between expression and genetic architecture, we sought to explore the effect of diet by comparing the *cis* and *trans* architecture of eQTL from separate genome scans of HFCA- fed mice (*n* = 134) and HP-fed mice (*n* = 109). The number of total eQTL found differed between diet groups, with 5596 eQTL in the HFCA-fed mice and 5002 in the HP-fed mice. We identified 3584 *cis*-eQTL and 2012 *trans*-eQTL in the HFCA-fed mice and 3254 *cis*-eQTL and 1748 *trans*-eQTL in the HP-fed mice (Table 2). The relative proportion of *trans*- eQTL was similar between diets: ∼36% of eQTL were *trans*- acting in the HFCA diet model results, compared to 35% in the HP diet model results. The *cis*-eQTL of the HFCA- and HP-fed mice overlapped by 65% and 66%, while the *trans*- eQTLs overlapped by 4.6 and 5.3%, respectively (Figure 7, A–F). mirQTL analysis within the HFCA and HP diets, using individually permuted significance thresholds, yielded few mirQTL. There were 19 significant mirQTL in the HFCA diet, 9 (47%) of which were *cis*-mirQTL, and 30 significant mirQTL in the HP diet, only 9 (30%) were a *cis*- mirQTL. Overlap analysis revealed that 4 *cis*-mirQTL were represented in the HP diet and the HFCA-fed mice. Conversely, the HFCA- and HP-fed mice overlapped by a single *trans*-mirQTL.

We then classified the eQTL results from the diet-specific QTL analysis as to their status relative to the differential expression results. Similar to the trend observed in the additive model, mRNA and miRNA were less likely to have an eQTL or mirQTL when they were differentially expressed (Supplementary Tables S10 and S11). Furthermore, the odds of an eQTL having a differentially expressed gene were similar between HFCA and HP diet models (HFCA diet: 0.83, 95% CI 0.76–0.91, *P < *0.001; HP diet: 0.78, 95% CI 0.71–0.86, *P *<* *0.001). To further explore the role of diet on *cis* and *trans* architecture, we calculated the phenotypic variance explained by eQTL and mirQTL in each diet model. In both HFCA-fed mice and HP-fed mice, the median variance explained for *cis*-eQTL was similar, at 0.282 and 0.295, respectively. *Trans*-eQTL explained 0.09 of the variance in the HFCA-fed mice and 0.08 in the HP-fed mice (Supplementary Figure S3). This result follows the trend observed in the additive model that *cis*-eQTL explain more variance than *trans*-eQTL; however, the variance explained from the diet models is greater than that of the additive model, suggesting an environmental perturbation effect.

**Figure 8:**
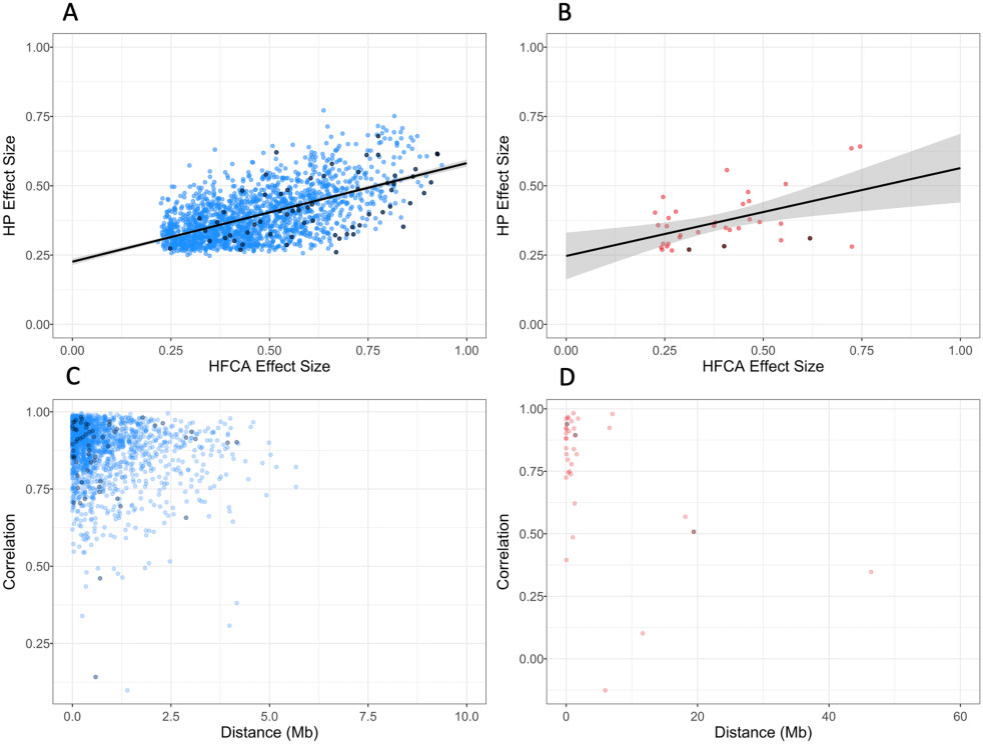
Comparison of concordant eQTL pairs in mice fed different diets demonstrates similarities despite different environments. Effect size of eQTL observed in both HFCA and HP diets are significantly correlated in both (A) *cis*-eQTL (*r *=* *0.99) and (B) *trans*-eQTL (*r *=* *0.99). Allele effects underlying concordant eQTL are highly correlated in both (C) *cis*-eQTL and (D) *trans*-eQTL despite different diet perturbations. Black dots represent eQTL with a significant allele-diet interaction (ANOVA, BH-adjusted *P *<* *0.05). Color gradient represents dot density.

Overall, 33% of eQTL in the HFCA-fed mice were replicated in the HP-fed mice. This corresponded to 2680 eQTL in total, which were predominately *cis*. We identified 2342 concordant *cis*-eQTL and 166 concordant *trans*-eQTL overlapping between models. Power differences between the HFCA- and HP-fed mice resulted in differences in resolution and led to different classifications of 172 eQTL (Figure 7, E and F), guiding our choice to only discuss eQTL (*n *=* *2680) that agreed in their classifications. We hypothesized that concordant eQTL would have similar effect sizes despite the different diets. The correlation of effect size between HFCA and HP diet models is 0.99 for both *cis*-eQTL and *trans*-eQTL (Figure 8, A and B), suggesting a congruent relationship. Next, we calculated the effects of the eight founder strains at the peak SNP for each eQTL, using BLUP coefficients in each diet model. We correlated the allelic effects and assessed how their correlation related to the distance between the peak SNPs of the eQTL (Figure 8, C and D). We observed that allele effects underlying eQTL were highly correlated in concordant eQTL in both *cis*-eQTL and *trans*-eQTL. We previously identified that alleles from the wild-derived strains CAST/EiJ and PWK/PhJ significantly contributed to eQTL and mirQTL in the full cohort with diet as an additive covariate. In both HFCA and HP diet models, CAST/EiJ and PWK/PhJ remained prominent contributors to eQTL and mirQTL, despite reduced power as a result of dichotomizing the cohort by diet (Supplementary Figure S4).

We next investigated the relationships between mRNA and miRNA in each diet subset and established the median absolute magnitude of the miRNA-mRNA correlations. Of all correlations, miRNA: *trans*-eQTL in the HFCA diet had the highest median absolute Spearman’s rho of 0.249 ± 0.00032, suggesting a coordinated response between the RNA classes in the extreme HFCA diet. The median value for *cis*-eQTL and no-eQTL were similar at 0.232 ± 0.0019 and 0.232 ± 0.0001, respectively. In the HP diet, the median rho for *trans*, *cis*, and no eQTL were similar at 0.235 ± 0.00025 and 0.235 ± 0.0003, and 0.234 ± 0.0001 (Supplementary Figure S5, A and B). We acknowledge that the number and distributions of *cis*- and *trans*-eQTL are different between the diets. Therefore, the differences in the correlation results between diets may in part be attributed to power.

### Gene-by-diet interactions reveal the significance of diet on eQTL and mirQTL

Given our previous reports of diet effects on hepatic gene expression (Coffey *et al.* 2017, 2019) and our observation of differing correlative structures between homologous GWAS candidate genes and traits in the DO population, we hypothesized that diet could affect the overall structure of eQTL and mirQTL. Thus, we performed eQTL and mirQTL analysis with diet as an interactive covariate and identified 6155 significant eQTL and 22 mirQTL (Supplementary Figure S6, A and B), after completing individual permutation thresholds (Table 1). Of these, 4861 eQTL were *cis*-acting and 2171 were *trans*-acting, while 12 mirQTL were *cis*-acting and 10 mirQTL were *trans*-acting (Table 2). As expected, the resolution of *cis*-eQTL and mirQTL identified in the interactive model was similar to the additive model, with the median distances between the TSS and the peak SNP’s physical location being 0.29 and 0.35 Mb, respectively.

Next, we compared the relative distribution of *cis*- and *trans*-acting eQTL in both the additive and interactive models. Overlap between significant eQTLs using diet as an interactive or additive covariate revealed large architectural differences in *trans*-eQTLs compared to *cis*-eQTLs. Large overlap between *cis*-eQTLs was observed between the models with 4723 transcripts with clear *cis*-acting regulation, and 84 and 98% of *cis*-eQTLs overlapping from the additive and interactive models, respectively. Conversely, only 37 and 36% of *trans*-eQTLs overlapped between the additive and interactive models (Supplementary Figure S6E). mirQTL architecture was also affected by the type of QTL model, as only 59 and 83% of *cis*-mirQTL were similar between the additive and interactive models while 23 and 30% of *trans*-mirQTL overlapped, proportions similar to trans-mirQTL (Supplementary Figure S6F). The sparse overlap of *trans*-eQTL may be due in part to their smaller effect sizes relative to *cis*-eQTL yet may suggest that genes regulated in *trans* are more sensitive to gene-by-diet interactions than those regulated by *cis* factors.

### Concordant eQTL are driven by different founder alleles in response to diet

**Figure 9:**
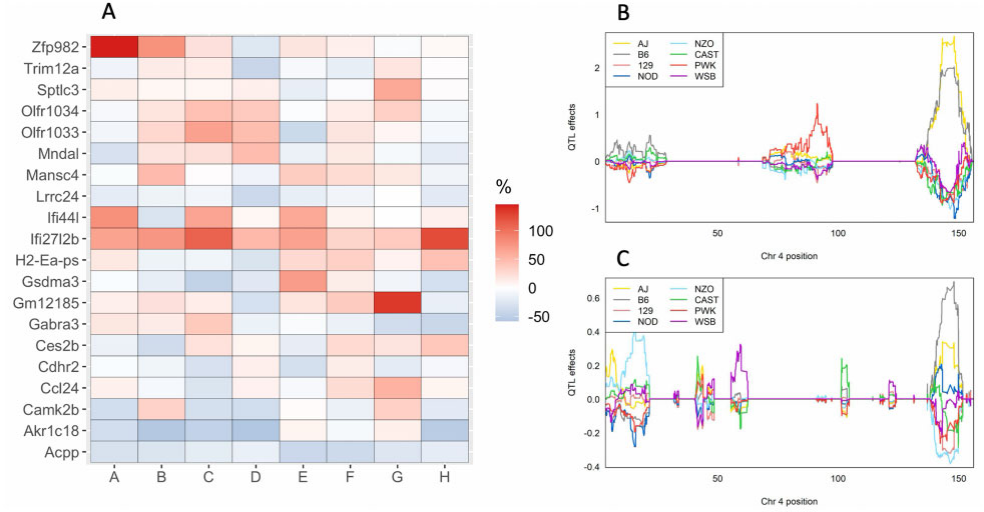
Significant genotype by diet interactions on the mRNA expression. (A) The top 20 genes with the greatest percent change of the best linear unbiased predictors (BLUP) founder allele coefficients for eQTLs with a significant allele-diet interaction in the HFCA diet with respect to the HP diet. (B) BLUPs coefficient plot of the eight founder mice strains to the *Ighm* eQTL in the HFCA diet and (C) the HP diet. Color represents the eight founder mice strains as indicated.

The genetic architecture differed between the additive and interactive models, indicating that a subset of eQTL could have a distinct response to diet. In support of this hypothesis, we identified that the allele effects of concordant eQTL in the HFCA and HP diet models were highly correlated; however, this was not universal as the correlation of the allele effects was often below one (Figure 8, C and D). We hypothesized that a subset of eQTL present in both the HFCA and HP diet models could be affected by alternative founder alleles. To test this, we began by identifying eQTL with significant allele- diet interactions in the interactive model. To accomplish this, we performed an ANOVA of the allele-diet interaction against the expression of the transcript with a significant eQTL. Of the 7032 eQTL identified in the interactive model, 1403 had a significant allele-diet interaction (*P *<* *0.05) and 308 were significant after BH correction (BH-adjusted *P *<* *0.05) (Supplementary Table S12). We then subset the eQTL present in both the HFCA and HP diet models to those with a significant (BH-adjusted *P *<* *0.05) allele-diet interaction, which represented 79 eQTL. We determined the effect of diet by calculating the percent difference in the founder allele effects of the HFCA-fed mice compared to the reference, HP-fed mice (Supplementary Table S13). We summed the absolute value of the change across all of the founders for each gene and highlight the top 20 in Figure 9A and provide an example (Figure 9, C and D). The *cis*-acting mRNA corresponding to Immunoglobin Heavy Constant Mu (Ighm) demonstrates a distinct response to diet; in the HP diet, the eQTL is driven by NZO/HILtJ and 129/SvJ, whereas in the HFCA diet, the same eQTL is driven only by NZO/HiLtJ (Figure 9, C and D).

## Discussion

GWASs have revolutionized genetic studies and expanded our understanding of complex trait genetics and genomic variants associated with disease susceptibility. Numerous studies have demonstrated that expression traits (eQTL) can be used as quantitative traits, and integrative studies of eQTL provide improved candidate selection at GWAS loci (Zhu *et al.* 2016). Despite these successes, much remains to be determined in regard to the genetic architecture. Identification of missing heritability (Edwards *et al.* 2013) and gene-by-environment interactions (Tan *et al.* 2012) are of particular interest. In this study, we reported the global regulation of hepatic mRNA and miRNA expression using a genetically diverse population of mice named the DO. Our studies identified several important results. First, we classified (*cis* and *trans*) the genetic regulation of mRNA and miRNA in DO mice and proceeded to characterize the eQTL and mirQTL by describing heritability, phenotypic variance explained, and allelic contributions. Second, by coupling our correlation and differential expression analyses, we showed increased correlation between miRNA-mRNA pairs differentially expressed by diet and most importantly increased correlation between miRNA- mRNA pairs without an eQTL. Finally, we reported that several eQTL are driven by different founder alleles as a result of diet. We discuss each of these in turn.

### Classification of genetic architecture of hepatic mRNA and miRNA

A primary goal of our study was to describe the genetic architecture of hepatic mRNA and miRNA expression profiles. Our initial analysis identified eQTL and mirQTL, using diet as an additive covariate. We classified these as *cis-* or *trans*-acting and used the *cis*-acting mirQTL and eQTL to estimate resolution. The DO mice have high-resolution mapping as demonstrated by the fact that 95–99% mirQTL and eQTL with *cis*-acting variants exert their effect within 1 Mb of the physical position of the mRNA or miRNA.

One of the key strengths of a “systems genetic” approach is the ability to resolve the genetic architecture of different scales of data such as mRNA and miRNA expression. In these studies, we identified thousands of genes regulated by eQTL but only dozens of miRNA regulated by mirQTL. The sheer difference in numbers between these two scales of data are notable but coincides with previous reports. The lower number of mirQTL has been previously reported in the Framingham cohort, where 76 miRNAs were regulated by mirQTL (Huan *et al.* 2015). We acknowledge that the differences (or lack of statistically significant differences) reported in subsequent analyses related to the distributions of eQTL and mirQTL could reflect the much smaller number of miRNA quantitated in this study.

Our analyses identified differing genetic architecture affecting mRNA and miRNA but confirmed that *cis*-acting variants affecting both mRNA and miRNA explain more phenotypic variance than *trans*-acting variants. These results suggest that miRNA are more likely to be regulated by *trans*- acting factors, while mRNA are more likely to be regulated by *cis*-acting factors. Over 50% of mirQTL are *trans*-acting, whereas <25% of eQTL are *trans*-acting. Unlike a recent report of eQTL in yeast, we do not observe that most eQTL are *trans*-acting in nature (Albert *et al.* 2018), but this could be due to differences in power or model organism of each study. While the functional consequences of this prominent difference in genetic architecture of mRNA and miRNA remain unclear, similar effects have been observed in studies utilizing CC mice (Rutledge *et al.* 2015), a genetically related population to the DO mice used in this study. Although speculative, these data from the CC and DO mouse populations suggest that individual miRNAs on average may be regulated by fewer transcription factors than individual genes. In such a scenario, SNPs affecting transcription factor loci would be expected to have greater effects on miRNA expression than on mRNA expression, which has multiple transcription factors regulating their expression.

Identifying the “optimal” definition of *cis* and *trans* (or local and distal) eQTL remains an open question. We note that we used a liberal definition of 4 Mb to define *cis*-eQTL, which reflects the mean confidence interval (∼4 Mb) for all significant eQTL, as determined by the Bayesian credible interval (see Materials and Methods). In addition, human GWASs have observed regions of SNPs that are physically located ∼2 Mb apart, in LD and are associated with a complex trait, such as height (Yang *et al.* 2012). For these reasons, we chose to utilize a larger genomic interval (4 Mb) to classify the eQTL. We mention that using a 1 Mb definition of *cis*, a similar window as human GWASs, would classify 923 additional eQTL, ∼14%, as *trans* instead of *cis*. Importantly, the classification used here and within many eQTL studies is computational and not based on functional testing. Recombination rate and local LD structures will affect any global definition of *cis* or *trans*. Regardless of the classification given to a particular eQTL, understanding the genetic variant’s contribution to differences in expression is ultimately the goal, but it is not yet feasible to test the mode of function or identify the causal variants for each eQTL.

Much emphasis has been placed on understanding the missing heritability observed in GWASs. Molecular traits provide a dense phenotypic space to explore these differences, and in our current studies, the median heritability of eQTL is 0.295 and mirQTL heritability is 0.24 in the additive model. The heritability estimates for eQTL are slightly higher than those recently reported in human blood cells, which was 0.089 (Lloyd-Jones *et al.* 2017), and may reflect the relatively similar environmental variation afforded by studies utilizing rodents. Our heritability estimates for mirQTL are quite similar to those previously reported in humans, where heritability of miRNA ranged between 0.0 and 0.57, with an average heritability of 0.11 (Huan *et al.* 2015; Lloyd-Jones *et al.* 2017). Similar to reports in humans, a subset of both eQTL and mirQTL in the DO population have heritability at or near 0.0 (Huan *et al.* 2015). Although the heritability of eQTL and mirQTL were similar, results from the additive model indicate that the effect size of eQTL varied considerably between 0.02 and 0.93, while mirQTL were limited to a range of ∼0.1–0.43. These differences in effect sizes were also evident when eQTL and mirQTL were characterized by their type of regulation, *cis*- or *trans*-acting. The phenotypic variance explained by *cis*- acting eQTL was 63% higher than *trans*-acting eQTL, and a similar trend was observed in mirQTL. Furthermore, these trends proved to be robust, as *cis*-acting eQTL explained more variance than their *trans*-acting counterparts in the diet-specific analyses.

Studies utilizing the DO population often assign probabilities at each SNP to the eight founder alleles (Gatti *et al.* 2014). Three of the founder strains, CAST/EiJ, WSB/EiJ, and PWK/PhJ, are wild-derived and are more divergent from classical inbred strains. These strains contain between 900, 1000, and 5 million private SNPs (Keane *et al.* 2011), and these are passed along at relatively equal proportions in the DO population (Chesler *et al.* 2016). Thus, the DO population contains variants that affect complex traits at loci that may not vary genetically in classical inbred strains (Keane *et al.* 2011). We hypothesized that variants from these divergent strains contribute disproportionally to eQTL and mirQTL. To test this hypothesis, we calculated the allele effects for each of the founder strains at the peak SNP for each gene or miRNA. Regardless of the model, the scaled coefficients for each founder haplotype were significantly higher for CAST/EiJ and PWK/PhJ alleles at each eQTL, indicating that a higher proportion of the variation in the expression of these eQTL and mirQTL are explained by variants from these strains. Similar effects have recently been reported in the related CC population (Keele *et al.* 2020). The authors also noted both higher magnitude of genetic effects for CAST/EiJ and PWK/PhJ alleles and relative consistency of haplotype effects derived from the eight founder strains. The exact mechanisms underlying these eQTL and the contribution of specific haplotypes remain to be determined, but are supported by large-scale resequencing. These efforts have included the eight founders of the DO and CC populations and identified a number of loci that contain novel genes or novel orthologs and are enriched for proteins associated with defense and immunity, nucleic acid binding, and transcription factors (Lilue *et al.* 2018). In particular, two of the founder strains of the DO and CC populations, CAST/EiJ and PWK/PhJ, contained a number of loci with high sequence variation, supporting the findings of the current study.

### Relationships between hepatic miRNA and mRNA are greater when diet affects expression

mRNA and miRNA have different genetic architecture, yet our data suggests similar correlation between mRNA and miRNA regardless of the underlying *cis* and *trans* regulation. More detailed examination of the effect of diet suggests that miRNA-mRNA pairs differentially expressed by diet were generally more correlated than pairs unaffected by diet regardless of the eQTL’s mapping status. These data support that miRNA-mRNA associations act as an additional regulatory mechanism underlying an organism’s response to the environment. This global perspective does not address direct interactions between mRNA and miRNA, which are important aspects of gene regulation. Approximately half of the significant correlations we observed were positive; these scenarios may be indicative of enriched pathways as opposed to interactions between miRNA and target mRNA, which would be predicted to have negative correlations. Clearly, a number of the correlations contained in the global analysis are indirect and coincidental. When miRNA-mRNA correlations are restricted to interactions validated in miRTarBase, we observe multiple interactions associated with cardiovascular and metabolic syndromes. For example, miR-34a is negatively correlated (Spearman’s rho = −0.760) with Autophagy Related 9 A protein, ATG9a, a gene previously shown to be involved in cardiomyocyte hypertrophy and regulated by miR-34a (Huang *et al.* 2014). miR-34a is also negatively associated with growth arrest specific 1, GAS1 (Spearman’s rho = −0.578), a GWAS candidate associated with plasma triglyceride levels (Rhee *et al.* 2013). We do acknowledge that we are more likely to detect QTL with inflated effect size and are unable to recognize every QTL due to power (King and Long 2017); however, within a specific diet condition, most miRNA-mRNA associations occur between miRNA and mRNA without an eQTL suggesting that coordination between the RNA classes occurs most in the absence of a strong genetic signal (Supplementary Tables S10 and S11). We also note that miRNA-mRNA regulatory networks are complex and could involve indirect effects outside of the classically described direct binding of a miRNA to a 3′UTR (Su *et al.* 2011).

### Diet has varying effects on eQTL and mirQTL

There is much interest in determining the missing heritability observed in GWASs. One possible cause of missing heritability is differences in environmental exposures, which implicitly vary in large GWAS analysis. More importantly, the effect of environment in these large genetic studies is heterogenous within the study population and often difficult if not impossible to accurately quantify. In this study, we varied diet in siblings and performed the initial analysis with diet as a covariate. To further understand the role of diet, we completed differential expression analysis of mRNA and miRNA by diet. The proportions of differential expression were similar between RNA classes, with 76% of genes and 80% of miRNA differentially expressed, suggesting similar responsiveness to diet. In addition, mRNA with a significant eQTL were less likely to be differentially expressed than nonmapping mRNA. In miRNA the pattern persisted but was not statistically significant. When we classified eQTL by their differential expression status, we clearly observed that phenotypic variance explained was greater in nondifferentially expressed genes, perhaps indicating a stronger genetic than environmental signal regulating their expression.

Inbred strains are known to vary in their response to diet (West *et al.* 1992), and thus it is quite possible that a fraction of eQTL are influenced by diet. There have been limited investigations characterizing how diet affects eQTL status. Con- somic mice have been used to confirm that alleles from AJ mice are responsible for *Xbp1* and *Socs3* diet-specific eQTL (Pasricha *et al.* 2015). Studies utilizing a different mouse population, the Hybrid Mouse Diversity Panel, have noted diet-specific eQTL patterns (Parks *et al.* 2013). In this study, we identified eQTL with a significant allele-diet interaction by regression analysis. This subset of eQTL represent genes whose founder alleles differ in response between diets. We further identified the top 20 genes whose allele effects were most dramatically changed by diet. For instance, we identified *Ighm*, immunoglobulin heavy constant mu, whose expression is associated with NZO/HILtJ and 129/SvJ alleles in the HP diet and only the NZO/HiLtJ allele in the HFCA diet. While the functional consequences of a gene-by-diet interaction for *Zfp982* remains to be determined, several of the genes with allele by diet interactions are GWAS hits. For example, variants proximal to *Camk2b* have been associated with numerous traits in humans, including diabetes (Morris *et al.* 2012). Functional studies in mice have demonstrated that *Camk2b*^−/−^ are susceptible to obesity (Bachstetter *et al.* 2014) and that the enzyme *Camk2b* is translated into, CaMKII, is involved in the hepatic insulin resistance that occurs with obesity (Ozcan *et al.* 2013). Furthermore, there is evidence that expression of Camk2b may also be under genetic regulation in humans (Frau *et al.* 2017; https://www.gtexportal.org/home/locusBrowserPage/ENSG00000058404.19). These results provide evidence that gene-by-diet interactions affect the mRNA abundance observed in the DO population and may have important implications for disease-related phenotypes.

Additional work remains to understand the underlying mechanisms regulating both differential gene expression and allele-diet interactions. Integration of multiple scales of sequencing data may provide additional insight. For example, assaying chromatin accessibility or methylation patterns could help understand the specific variants or DNA modifications by which diet exerts its effects on gene expression. Recently, both the genetic regulation of gene expression and chromatin accessibility (assay for transposase-accessible chromatin using sequencing) was assessed in 47 strains comprising the CC mouse panel (Keele *et al.* 2020). Keele and colleagues highlight the tissue specificity of eQTL, the underlying DNA modifications corresponding to specific eQTL, and perform mediation analysis to identify potentially causal variation in chromatin accessibility responsible for specific eQTL. Diet has been shown to affect chromatin structure (Leung *et al.* 2014, 2016) and thus provides a plausible connection between diet-driven DNA modifications and eQTL. Although not tested in this study, it is intriguing to speculate that some of the differential expression and diet-specific eQTL are due to changes in chromatin structure.

In conclusion, gene expression studies in a segregating population provide interesting insights into complex genetic regulation. In addition, assessing these patterns in the context of diet helps to reveal the relationships between genetics and the environment. In this study, we have classified the genetic regulation and characterized the genetic architecture of hepatic mRNA and miRNA in a genetically diverse population of mice fed different diets. We observed significant differences in the regulation, effect size, and overall genetic architecture of mRNA and miRNA, and note the varying effects of diet on these trends. Overall, these key regulatory differences underscore the necessity for continued investigation into how the diverse spectrum of RNA classes are regulated, how they respond to environmental stimuli such as diet, and how dysregulation may predispose an organism to disease.
